# Serine/threonine kinase 36 induced epithelial-mesenchymal transition promotes docetaxel resistance in prostate cancer

**DOI:** 10.1038/s41598-024-51360-9

**Published:** 2024-01-06

**Authors:** Tao He, Nan-Xing Li, Zhao-Jun Pan, Zi-Hao Zou, Jie-Chuan Chen, Si-Zhe Yu, Fa Lv, Quan-Cheng Xie, Jun Zou

**Affiliations:** 1 Department of Emergency Surgery, Guangdong Provincial Key Laboratory of Major Obstetric Diseases; Guangdong Provincial Clinical Research Center for Obstetrics and Gynecology; The Third Affiliated Hospital of Guangzhou Medical University, 63 DuoBao Road, Guangzhou, Guangdong 510150 People’s Republic of China; 2 Department of Urology, Guangdong Provincial Key Laboratory of Major Obstetric Diseases; Guangdong Provincial Clinical Research Center for Obstetrics and Gynecology; The Third Affiliated Hospital of Guangzhou Medical University, Guangzhou, Guangdong 510150 People’s Republic of China; 3 The Third Clinical College of Guangzhou Medical University, Guangzhou, Guangdong 511436 People’s Republic of China

**Keywords:** Cancer, Cancer therapy, Urological cancer

## Abstract

To investigate the role and potential mechanism of serine/threonine kinase 36 (STK36) in docetaxel resistance-prostate cancer (PCa). The expression of STK36 in PCa and the correlation with clinicopathological characteristics of PCa patients were analyzed using the data from different databases and tissue microarrays. To investigate the role of STK36 on cell proliferation, invasion, and migration, STK36 was overexpressed and silenced in DU-145 and PC-3 cell lines. Cell counting kit-8 (CCK8) was used to test cell proliferation. Cell invasion and migration were detected by cell wound scratch assay and trans well, respectively. The expression profile of STK36, E-Cadherin, and Vimentin was analyzed by Western blot. Cell apoptosis was detected by the TUNEL assay. STK36 expression was upregulated in PCa tissue compared with adjacent benign PCa tissue; it was higher in patients with advanced stages compared with lower stages and was significantly correlated with decreased overall survival. Up-regulation of STK36 significantly promoted the proliferation, invasion, and migration of DU-145 and PC-3 cells and compensated for the suppression caused by docetaxel treatment in vitro. A striking apoptosis inhibition could be observed when dealing with docetaxel, although the apoptosis of DU-145 and PC-3 cells was not affected by the STK36 exclusive overexpression. Besides, E-Cadherin expression was restrained while the expression levels of vimentin were all enhanced. The knockdown of STK36 reversed the above process. STK36 up-regulation could accelerate the biological behavior and docetaxel resistance of PCa by epithelial-mesenchymal transition (EMT) activation. STK36 may be potentially used as a target in PCa resolvent with docetaxel.

## Introduction

Prostate cancer is the second most commonly diagnosed cancer and the 5th cause of cancer-related death among men worldwide. The incidence and mortality of PCs in China are relatively low; however, the age-standardized incidence rate of PCs drastically increased from 1990 to 2019. Prostate cancer needs testosterone to grow. Androgen receptors have an important role in the development of PCa, and their activation can stimulate proliferation while inhibiting the apoptosis of PCa cells. Therefore, androgen deprivation therapy is the standard first-line treatment for advanced prostate cancer^1^. Castration-resistant prostate cancer (CRPC) is cancer that continues to grow even when the testosterone levels are at or below the castrate level^2^. Docetaxel is a semi-synthetic derivative of paclitaxel that can reduce the expression of androgen receptors on CRPC cells and is currently considered a relatively effective chemotherapy drug for prostate cancer^3,4^. However, drug resistance occurs in most patients during treatment, and the mechanisms therein have been only partially explored.

Many transcription factors and signaling pathway-related proteins are closely related to docetaxel. In tumor cells, docetaxel can bind their beta subunits of tubulin in microtubules, inhibit depolymerization, block mitosis, and induce apoptosis^5^. Docetaxel is also associated with changes in transcription levels of B-cell lymphoma-2 (Bcl-2)^6^, notch and hedgehog-dependent tumor-initiating (7), abnormal activation of P53^8^, nuclear factor kappa-B (NF-κB)^9^, phosphatidylinositol 3 kinase (PI3K)-Akt^10^, and other cell signaling pathways. Previous studies have shown that over-expression of microRNA (miRNA)-21 can increase the dependence of PCa cells on docetaxel^11^. Moreover, other invasion-related genes like histone demethylase lysine-specific demethylase 5D (KDM5D) can affect the sensitivity of docetaxel by interacting with the androgen receptor (AR) to change their transcriptional activity^12^. In addition, giant polyploid (MP) and autophagy are also deeply related to the generation and process of docetaxel resistance^13,14^.

The aggressive behavior of cancer cells resulting from EMT-mediated metastasis can also lead to docetaxel resistance^15^. EMT via the hedgehog signaling pathway (HSP) has been suggested as one of the mechanisms of the progression of CRPC^16^. E-Cadherin and vimentin are important markers in the process of epidermal mesenchymal transition, closely related to drug resistance and malignant progression of prostate cancer^17^. Also, some studies have shown that HSP, androgens, and EMT are closely related to PCa progression^18^. Inhibiting the HSP through small-molecule SMO inhibitors has shown effectiveness in pre-clinical CRPC models^19,20^. The activation of the HSP and cAMP-dependent protein kinase A (PKA) can be affected by STK36, which is synergized with glioma-associated oncogene protein (Gli1) and Gli2 to activate the HSP and can rescue HSP defects in zebrafish caused by the loss of zebrafish Fu homolog^21,22^. In gastric cancer, STK36 mediates the SMO activation by HSP, which leads to phosphorylation of Suppressor of Fused (SUFU) and the nuclear accumulation of full-length Gli family members by preventing nuclear export of Gli family members^23^. Involuntary, these deserve a suspicion that STK36 probably conserves a potential correlation with docetaxel, especially under the state that its function is still not clear in prostate cancer. In this study, we investigated the role and mechanism of serine/threonine kinase 36 (STK36) in docetaxel-resistant PCa.

## Materials and methods

### Cell culture

The focus of this article is to study CRPC chemotherapy resistance, so we selected two hormone resistant cell lines DU145 and PC3 as the research subjects.The human prostate cancer cell lines PC-3 and DU-145 were purchased from the Cell Bank of Shanghai Life Science Institution, Chinese Academy of Sciences and grown in Roswell Park Memorial Institute (RPMI) 1640 media supplemented with 10% fetal bovine serum (FBS) (Gibco, Carlsbad, CA, USA) and 1% streptomycin/penicillin. in a humidified atmosphere containing 5%CO_2_/95% air at 37ºC (Thermo Scientific, Waltham, MA, USA). The docetaxel (51,148, Selleck, Shanghai, China) concentration used to treat PC-3 and DU-145 was 0.1 μmol/L and 5 nmol/L, respectively.We have confirmed that the note of caution stated in Cellosaurus(https://www.cellosaurus.org/CVCL_0035) for the PC-3 cell line will not have an impact on our study.

### Overexpression and knock-down of STK36 in vitro

The STK36 overexpression construct was obtained by sub-cloning polymerase chain reaction (PCR). STK36 was amplified from the cDNA ORF clone (Sino Biological, Beijing, China) into the pcDNA 3.1 vector (Invitrogen, Carlsbad, CA, USA). The small interfering RNA (siRNA) sequences for STK36 were designed as follows: 5'-GACGUUGCUACUCUCUUUAdTdT-3'; negative control siRNA : 5’-UUCUCCGAACGUGUCACGUTT-3'. Starting from the AUG start codon of the STK36 transcript, "AA" binary sequence was searched, and its 19 base sequences at the 3 'end were recoded to design siRNA. siRNA of STK36 was synthesized by chemical synthesis method. siRNAs were transfected into cell lines DU-145 and PC-3 by utilizing Lipofectamine 3000 (Invitrogen, Carlsbad, CA, USA) following the manufacturer's instructions.

### Western blot

We are studying the protein function of STK36, not mRNA. Therefore, we do not conduct Q-PCR testing. The total protein was extracted using SDS Lysis Buffer (P0013G, Beyotime, Shanghai, China) and measured using a BCA Protein Assay Kit (Beyotime Biotechnology, Shanghai, China). The proteins were split by a 10% SDS polyacrylamide gel electrophoresis (PAGE) gel and then blotted onto a polyvinylidene fluoride (PVDF) membrane. Next, the membranes were blocked with 5% skim milk powder in phosphate-buffered saline-tween (PBST) for 1 h at room temperature (RT) and hatched with the specific primary antibody at 4 °C overnight. The primary antibodies used were anti-STK36 (1:1000, 12,559–1-AP, proteintech), anti-E Cadherin (1:1000, 20,874–1-AP, proteintech, chicago, USA), anti-Vimentin (1:1000, 10,366–1-AP, proteintech, chicago, USA), anti-N-Cadherin(1:1000,13,116,CST,Boston,USA), anti-β-Catenin (1:1000,8480,CST,Boston,USA), anti-Slug (1:1000,9885,CST,Boston,USA) and anti-glyceraldehyde-3-phosphate dehydrogenase (GAPDH) (1:1000, 10,494–1-AP, proteintech, chicago, USA). The membranes were washed with PBST and incubated with corresponding horseradish peroxidase (HRP) Goat anti-Mouse IgG (1:5000, 15,014, Proteintech, Chicago, USA) for 30 min at 37 °C. Densitometric analysis was performed using ImageJ (National Institutes of Health, Bethesda, MD, USA), and values were normalized to GAPDH.The amount of protein that was loaded per lane in SDS-PAGE is 20ug. The bands were visualized in western blotting using ECL(32,106,Thermofisher,Massachusetts,USA).

### Immunohistochemical (IHC) analyses

The expression of STK36 was assessed using IHC on tissue PCa microarrayanalysis as previously described^24^. The chip was purchased from Zhongke Guanghua (Xi'an, China) Intelligent Biotechnology Co. PCa tissues and adjacent noncancerous tissues from 79 cases confirmed with PCa were selected. Samples were stained slides that were digitally scanned using the T3 ScanScope (Aperio Technologies,Vista, CA) with a resolution of 0.5 mm/pixel. Each core was scored for the percent area positivity. The percentage of positive staining in each of the three cores from an individual case was averaged to derive a composite score of percent area positivity for each case, with at least 5% being considered positive. In addition, scanned images were aligned for all stains, and similar areas of focal positivity were noted. The anti-STK36 antibody (1:100, 12,559–1-AP, Proteintech) was used at 4 °C overnight before samples were treated with streptavidin–horseradish peroxidase complex (1:2000, A0303, Beyotime, Shanghai, China). Samples were stained slides that were digitally scanned using the T3 ScanScope (Aperio Technologies,Vista, CA) with a resolution of 0.5 mm/pixel.

### Cell proliferation

Cell proliferation was evaluated by CCK-8 (Beyotime Biotechnology, Shanghai, China). The PC-3 and DU-145 cells (1 × 10^4^/mL) were inoculated into 96-well plates and cultured for 48 h with 3 multiple wells for each group. Cells were then exposed to docetaxel (0.1 μmol/L for PC-3 and 5 nmol/L for DU-145) of docetaxel (treatment group) or PBS (control group) for 48 h. Then, 100 μl of CCK-8 reagent (10 mg/mL) was added to each well and incubated for another 4 h at 37 °C. The absorbance at 450 nm was determined using a microplate reader (100x). In addition, cell proliferation was detected by EDU assay using an EdU Kit (C10310-1, RiboBio,guangzhou,China) referring to the manufacturer’s instructions. Briefly, cells were incubated with 50 μM EdU per well for 2 h at 37 °C. Afterwards, the cells were fixed for 20 min at room temperature using 100 μl 4% polyformaldehyde. After permeabilization with 0.3% Triton for 7 min, the cells were incubated with 1X Apollo solution for 30 min at 37 °C in the dark. After incubated with 100 μl of 1X Hoechst 33,342 solution for 20 min at 37 °C in the dark followed, the cells were then visualized under a fluorescence microscopy.

### Cell wound scratch assay

The concentration of the cells was adjusted in the logarithmic phase and was inoculated into a 24-well plate with 500 μL 2.0 × 10^6^/mL. After the cell reached 90% confluence, a line was drawn using a marker on the bottom of the dish, and then a sterile 200 μL pipet tip was used to scratch three separate wounds through the cells, moving perpendicular to the line. Next, the cells were gently rinsed twice with PBS to remove floating cells and incubated in RPMI 1640 medium containing serum-free in 37 °C, 5% CO2/95% air environment. Images of the scratches were taken by using an inverted microscope at × 10 magnification at 0 and 24 h of incubation. The experiment was repeated 3 times, and the calculation was closed each time rate to reflect the cell's ability to migrate.

### Cell migration and invasion

Cell migration and invasion assays were performed using a modified transwell chamber (8um) migration assay and invasion assay matrigel-coated membrane (BD Biosciences Bedford, MA) as previously described^25^. Briefly, 1 × 10^4^ cells were suspended in serum-free medium, plated in 24-well plates, and treated with the indicated STK36 plasmid or STK36-siRNA with drugs. After 24 h, cell migration was measured as per the manufacturer’s instructions, while cell invasion was measured 48 h post-treatment, following the manufacturer’s instructions. Dr. Zou dyed Crystal violet in Fig. 5A overnight, and Dr. Li dyed Crystal violet in Fig. 3A overnight. Both experiments have been repeated Threefold repetition. The depth of Crystal violet dyeing does not affect the experimental results.

### Cell apoptosis

Cell apoptosis was detected by TdT-mediated dUTP nick-end labeling (TUNEL) assay as previously described^26^. Cells were fixed with 4.0% paraformaldehyde, permeabilized with 0.1% Triton X-100 in PBS for 15 min, and blocked in 3% H_2_O_2_-methanol. The cells were treated with proteinase K for 30 min at 37 °C, and then incubated with fluorescein-dUTP in the dark. The nuclei were counterstained using 4',6-diamidino-2-phenylindole (DAPI, YT891, Aolaibo, Beijing, China). TUNEL staining was observed under a confocal laser scanning microscope (LSM 510, Zeiss, Gottingen, Germany) and photographed at a magnification of 100 × . After washed three times with ice-cold PBS, cells were incubated with FITC-Annexin V and PI (abs50001, Absin, Shanghai, China ) for 20 min in the dark, and detected to flow cytometric analysis (Accuri C6, New Jersey, BD, USA ).

### Statistical analysis

Two prostate cancer datasets (GSE46602^27^ and GSE21032^28^) downloaded from Gene Expression Omnibus(http://www.ncbi.nlm.nih.gov/geo). Download the prostate cancer dataset with detailed clinical information from The Cancer Genome Atlas( https://xenabrowser.net/datapages/?cohort=TCGA%20Prostate%20Cancer%20(PRAD)&removeHub=https%3A%2F%2Fxena.treehouse.gi.ucsc.edu%3A443). The survival rate was calculated by the Kaplan–Meier method using log rank test and the Tarone Ware test. SPSS 18.0 software was used for data analysis (SPSS, Inc., USA). The results were presented as mean ± standard deviation (SD). One-way ANOVA was used to perform analysis of variance. Significant differences were identified using factorial analysis of unpaired student's t-test (two groups) and analysis of variance (multiple groups). P < 0.05 represented statistical significance and is indicated by an asterisk in the bar chart. All experiments were repeated 3 times.

### Ethics approval and consent to participate

These procedures were approved by the Research Ethics Committee of The Third Affiliated Hospital of Guangzhou Medical University (Guangzhou, China). The approval number is No [2023] 195.

## Results

### Upregulation of STK36 in prostatic cancer correlated with poor survival

**Figure 1:**
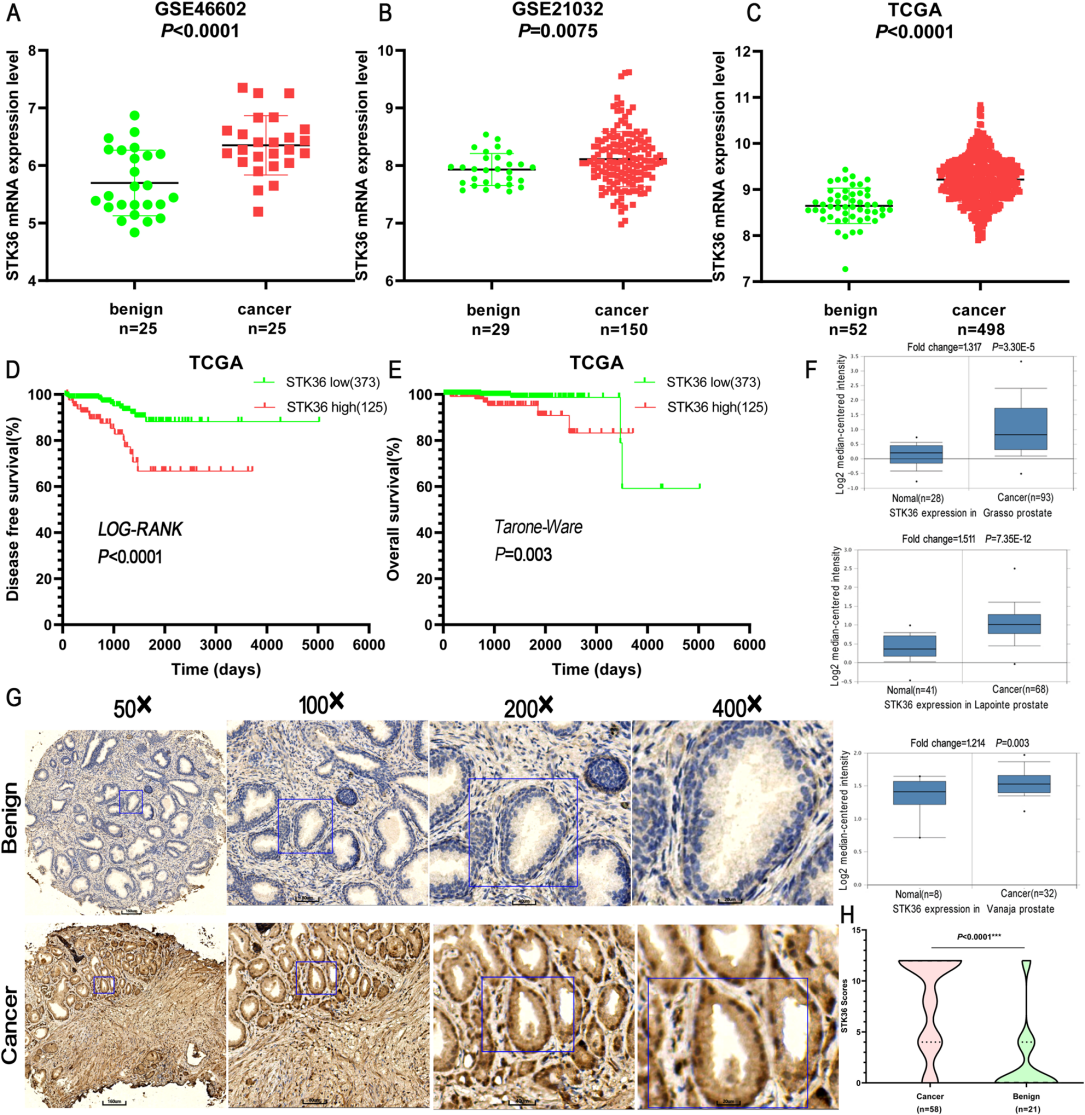
Upregulation of STK36 in prostatic cancer is correlated with poor survival. **(A-C)** STK36 mRNA expression levels were increased in PCa tissues compared to benign tissues from the three GEO and TCGA expression profiling datasets. **(D-E)** From TCGA PCa specimen cohorts, patients with high mRNA expression of STK36 had higher RFS and shorter OS compared to the patients with low STK36 expression. **(F)** STK36 mRNA expression levels were increased in PCa tissues compared to benign tissues from Oncomine profiling datasets. **(G-H)** IHC analysis of STK36 protein expression in benign tissues and PCa tissues. The whole scanned image of the tissue array has been uploaded as Supplementary Fig. 1.

The corresponding information from multiple databases was loaded to explore the association of STK36 expression with PCa. By analyzing the GSE46602^27^, GSE21032^28^, and the cancer genome atlas (TCGA) datasets, STK36 mRNA levels were significantly increased in PCa tumor tissue samples from patients (Fig. 1A–C). Consistently, after dividing the TCGA dataset into low and high STK36 expression groups according to the median value of STK36 mRNA levels, patients with high STK36 expression levels exhibited decreased disease-free survival compared to those with lower STK36 expression (log-rank test, Fig. 1D). Also, high STK36 expression was significantly correlated with decreased overall survival (Tarone Ware test, Fig. 1E). Similar results were observed in the samples obtained from the Oncomine database (Fig. 1F). Furthermore, an extensive tissue microarray analysis of PCa tissues and nontumor samples was performed using IHC with anti-STK36 antibodies (Fig. 1G). The expression of STK36 was positively correlated with pathological pattern (P < 0.0001), Gleason score (Homogeneity of variance test P-value 0.717, one-way ANOVA *P* = 0.027), and tumor Grade (*P* = 0.044) (Table 1). Besides, increased STK36 levels were observed in PCa tissues compared with the levels in adjacent nontumor samples using unpaired student's t-test (Fig. 1G,H).

**Table 1:** The relationship between the expression of STK36 protein in PCa tissue microarray and the clinicopathological characteristics of PCa.

Clinicopathological characteristics	n*	IHC score	*P *value
Pathological type			
Benign	21	1.90 ± 3.71	< 0.0001
Cancer	58	8.21 ± 4.58	
Age(year)			
< 60	6	6.00 ± 4.20	0.216
≥ 60	52	8.46 ± 4.20	
Gleason score			
≤ 6	12	6.00 ± 4.67	
7	5	4.80 ± 5.22	0.027
≥ 8	39	9.13 ± 4.20	
Grade stage			
I + II	21	6.48 ± 4.64	0.044
III + IV	35	9.03 ± 4.38	
TNM stage			
≤ T_2_	34	7.53 ± 4.80	0.183
≥ T_3_	24	9.17 ± 4.17	

*Two of the benign specimens were normal prostate tissue, while the rest were benign prostatic hyperplasia tissue. Some specimens of malignant tissue have missing data, only 56 specimens of 58 malignant tissues have Gleason score and Grade stage information.

### STK36 promotes PCa proliferation and decreases its sensitivity to docetaxel

**Figure 2:**
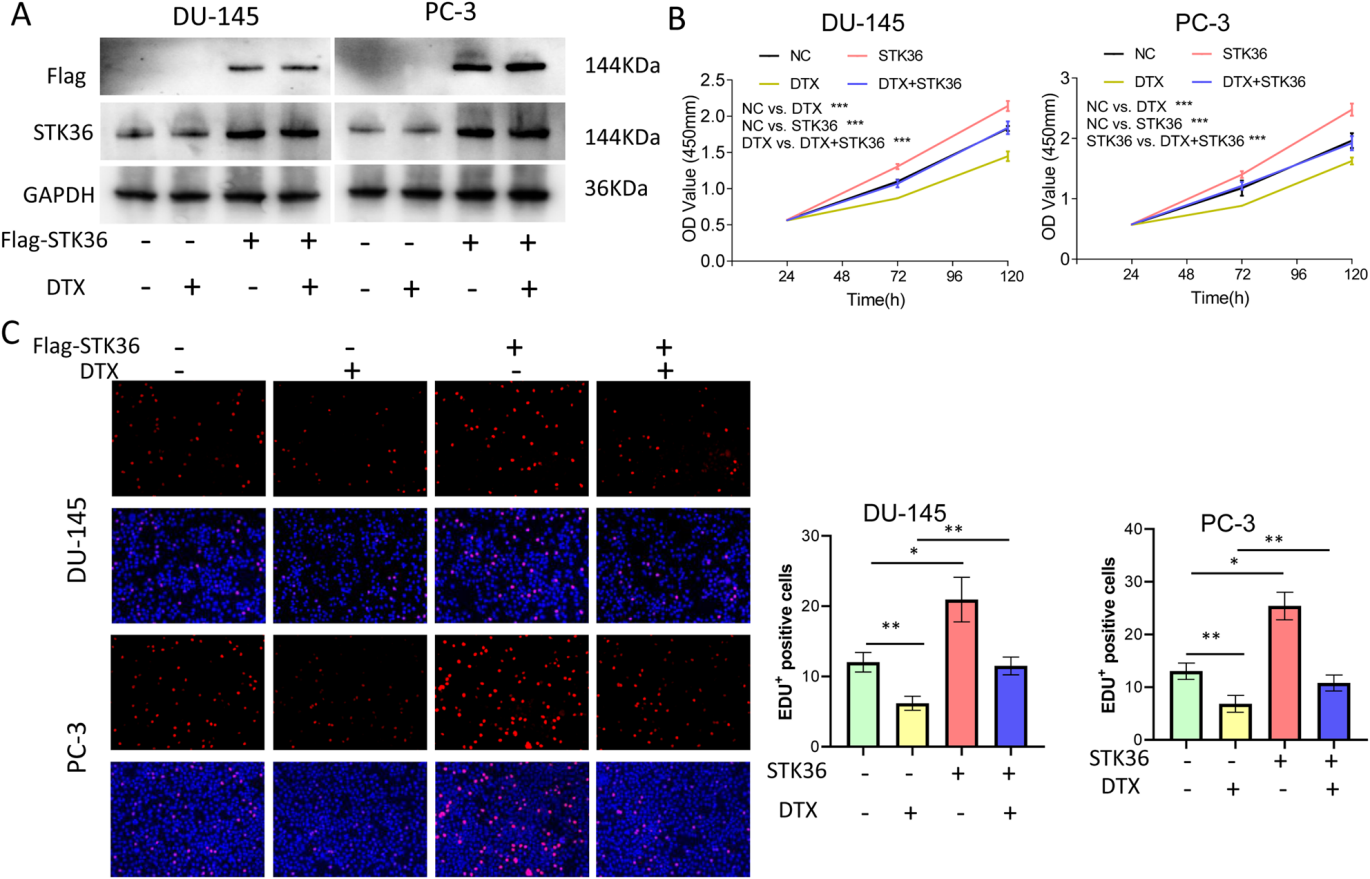
STK36 promotes PCa proliferation and decreases its sensitivity to docetaxel. **(A)** The docetaxel-treated concentration for PC-3 was 0.1 μmol/L, while it was 5 nM for DU-145. STK36 overexpression was detected by Western blotting. The bands shown in revised Fig. 2A derived from the same sample was confirmed. **(B, C)** Treated cell proliferation was evaluated by CCK-8 assay for 120 h and EDUassay for 48 h.

To investigate the role of STK36 on docetaxel-resistant to PCa, STK36 was primarily overexpressed in DU-145 and PC-3 cell lines using westernblot (Fig. 2A). Overexpression of STK36 significantly promoted the proliferation of both DU-145 and PC-3 cell lines compared to the NC group. Furthermore, cells proliferation was significantly inhibited with docetaxel treatment (Fig. 2B). When STK36 was overexpressed and simultaneously treated with docetaxel, the proliferation of DU-145 and PC-3 cells was significantly increased compared with the docetaxel treatment group (Fig. 2B, C).

### STK36 promotes migration and invasion of PCa cells at the expense of lower docetaxel sensitivity

**Figure 3:**
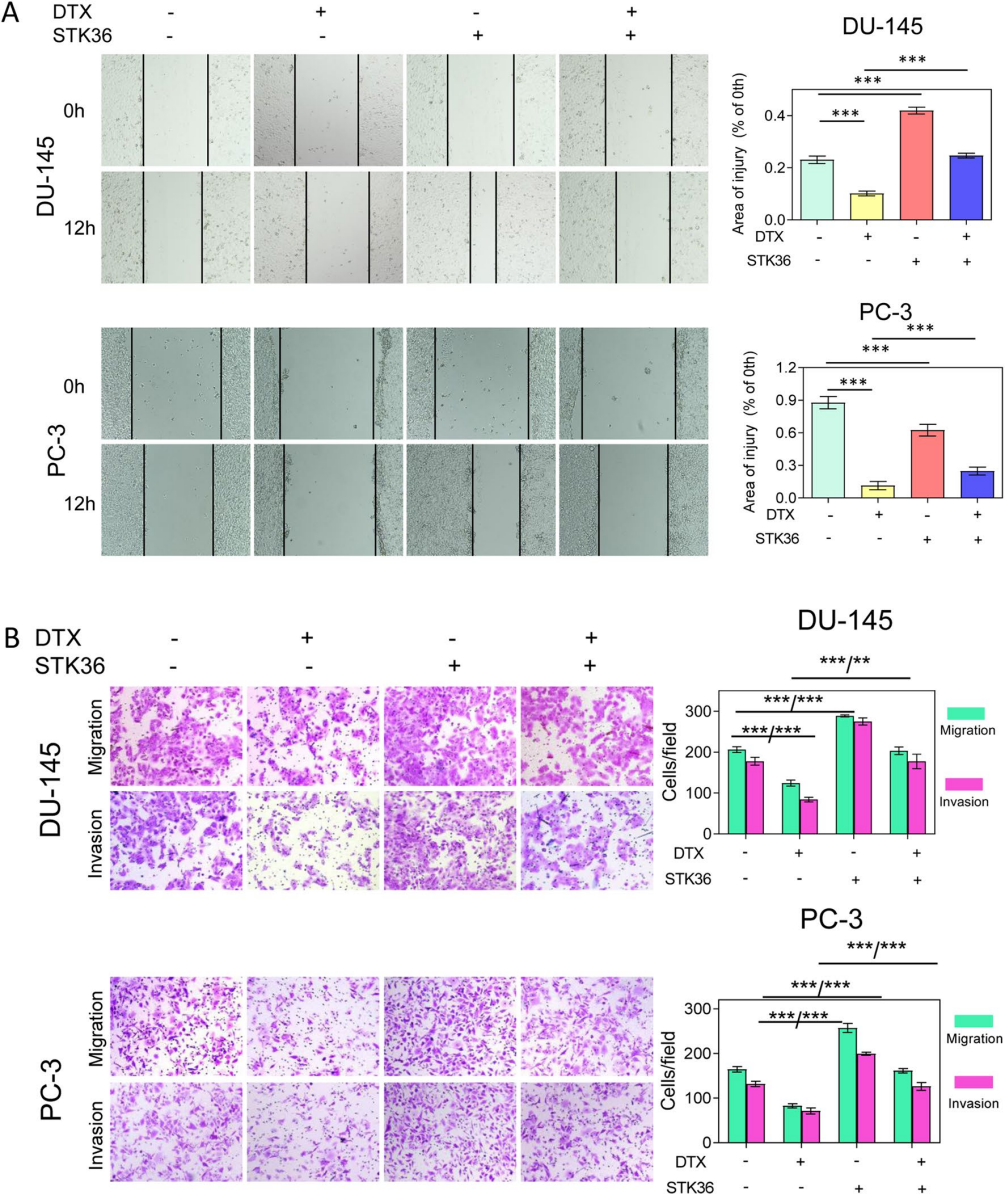
STK36 promotes distal metastasis of PCa cells at the expense of lower docetaxel sensitivity. (A) The docetaxel-treated concentration for PC-3 was 0.1 μmol/L, while it was 5 nM for DU-145. STK36 was over-expressed in DU-145 and PC-3 cells. The treated cells' concentration was adjusted in the logarithmic phase and was inoculated into a 24-well plate with 500 μL 2.0 × 10^6^/mL. Cell wound scratch assay was performed **(B).** 1 × 10^4^ cells suspended in serum-free medium were plated in 24-well plates and treated with the indicated STK36 plasmid with docetaxel. 24 h after treatment, cell migration and invasion were measured.

The migration and invasion of PCa cells were further examined. Exogenous overexpression of STK36 enhanced migration and invasion compared to the NC group, while docetaxel treatment reduced this effect (Fig. 3A,B). When STK36 was overexpressed and simultaneously treated with docetaxel, the inhibitory effect of the docetaxel could be blunted, and the migration and invasive capabilities of the cells were significantly improved compared to the docetaxel treatment group (Fig. 3A, B).

### STK36 down-regulation inhibits the proliferation and increases the docetaxel sensitivity of PCa cells

**Figure 4:**
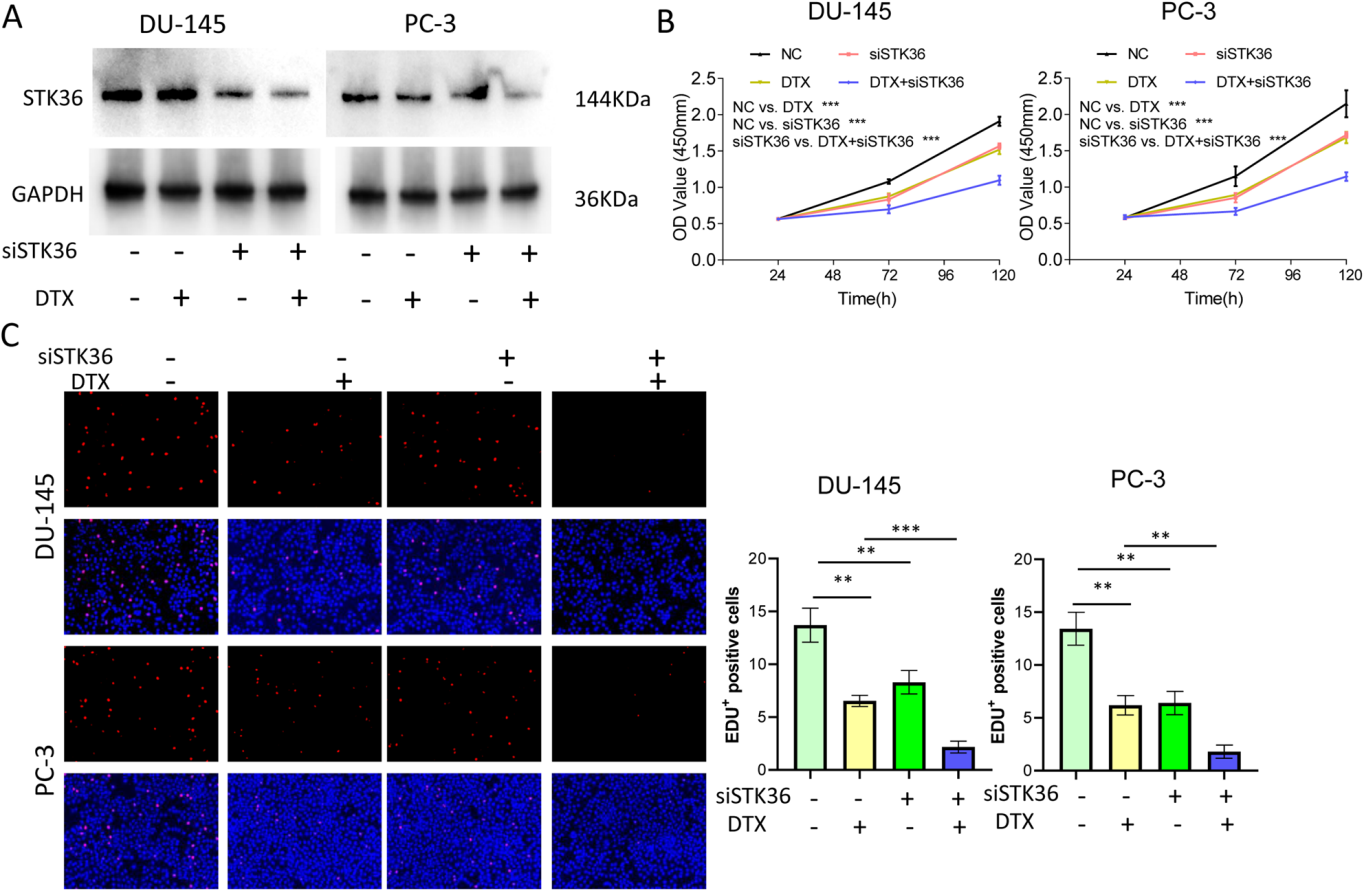
STK36 down-regulation inhibits the proliferation and increases docetaxel sensitivity of PCa cells. **(A)** The docetaxel-treated concentration for PC-3 was 0.1 μmol/L, while it was 5 nM for DU-145. STK36 overexpression was detected by Western blotting. (**B**, **C**) Treated cell proliferation was evaluated by CCK-8 assay for 120 h and EDU assay for 48 h.

To ensure the function of STK36 in docetaxel resistance of PCa cells, STK36 was inhibited using westernblot (Fig. 4A). After knock-down, the proliferation of DU-145 and PC-3 cell lines decreased compared to the NC group. The same results could be observed when cells were treated with docetaxel compared with the NC group (Fig. 4B, C). When STK36 down-regulation and docetaxel occured as superposition, the descending was further aggravated (Fig. 4B, C).

### STK36 down-regulation restrains the migration and increases the docetaxel sensitivity of PCa cells

**Figure 5:**
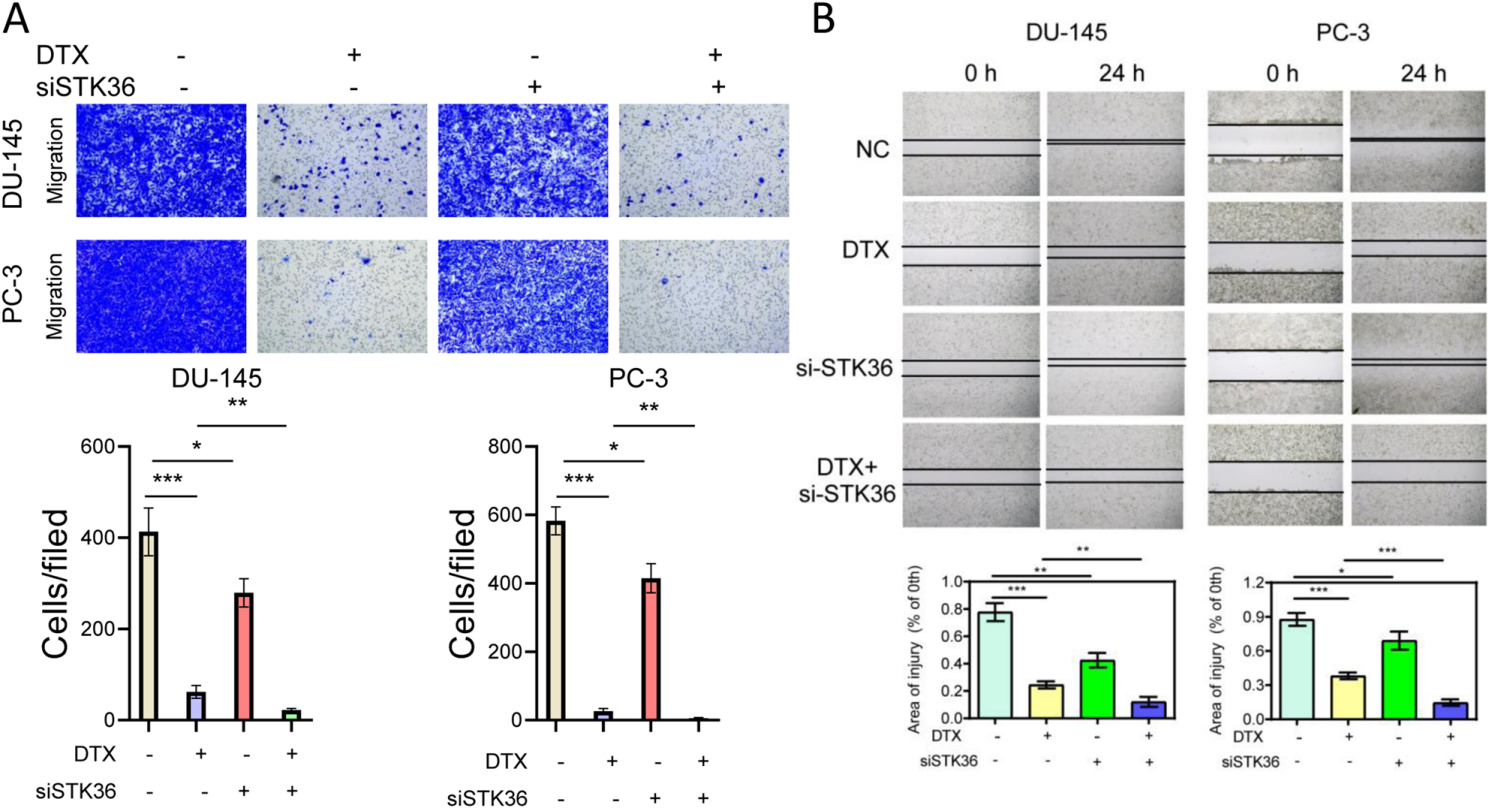
STK36 down-regulation restrains the migration but increases the docetaxel sensitivity of PCa cells. (**A**)The docetaxel-treated concentration for PC-3 was 0.1 μmol/L and for DU-145 was 5 nM, STK36 was knock-down in DU-145 and PC-3 cells.1 × 10^4^ cells suspended in serum-free medium were plated in 24-well plates and treated with the indicated STK36 plasmid with docetaxel. 24 h after treatment, cell migration and invasion were measured.(B)The treated cells concentration were adjusted in the logarithmic phase and was inoculated into a 24-well plate with 500 μL 2.0 × 10^6^/mL. Cell wound scratch assay was performed.

In order to contrast the cell peculiarity variations, proliferation, migration, and invasion of PCa cells under lower STK36 expression state were detected subsequently. The results of transwell and cell wound scratch all manifested that STK36 knock-down could motivate the migration of DU-145 and PC-3 cell lines, while the docetaxel showed an opposite effect compared to the NC group (Fig. 5A,B). When STK36 was knock-down and simultaneously treated with docetaxel, cell migration of the former was obviously inferior.

### STK36 subdued the docetaxel sensitivity and the proapoptotic effect of PCa cells

**Figure 6:**
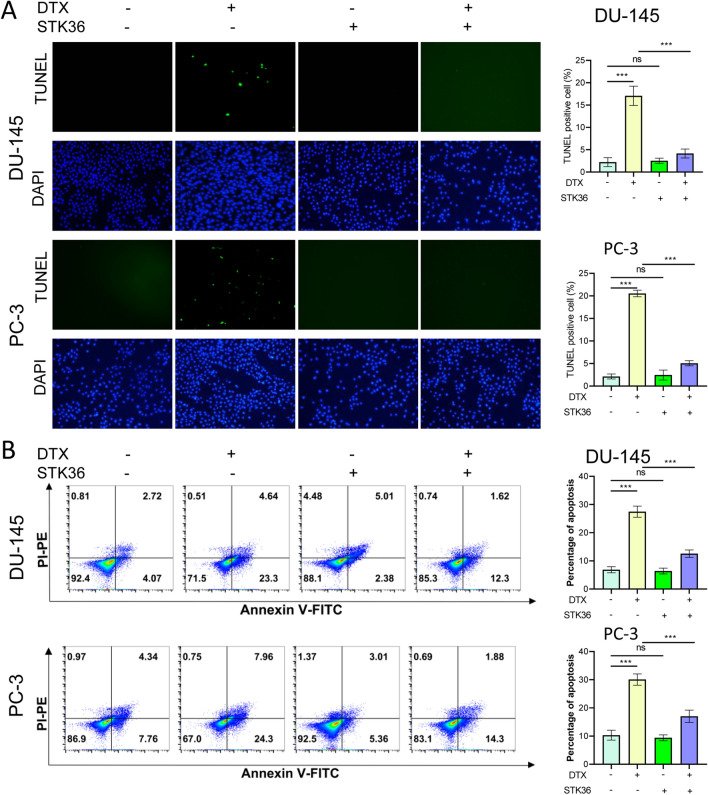
STK36 subdued the docetaxel sensitivity and the proapoptotic effect of PCa cells. (**A**, **B**)The docetaxel-treated concentration for PC-3 was 0.1 μmol/L, while it was 5 nM for DU-145. STK36 was overexpressed in DU-145 and PC-3 cells. Cell apoptosis was detected by tunnel assay and flow cytometry.

According to the cell apoptosis examination results, overexpression of STK36 had no impact. However, docetaxel treatment significantly accelerated the apoptosis of DU-145 and PC-3 cell lines (Fig. 6A, B). When STK36 was overexpressed and treated with docetaxel simultaneously, the apoptosis of DU-145 and PC-3 cells were both significantly reduced.

### STK36reduces the sensitivity of PCa cells to docetaxel by regulating epithelial-mesenchymal transition

**Figure 7:**
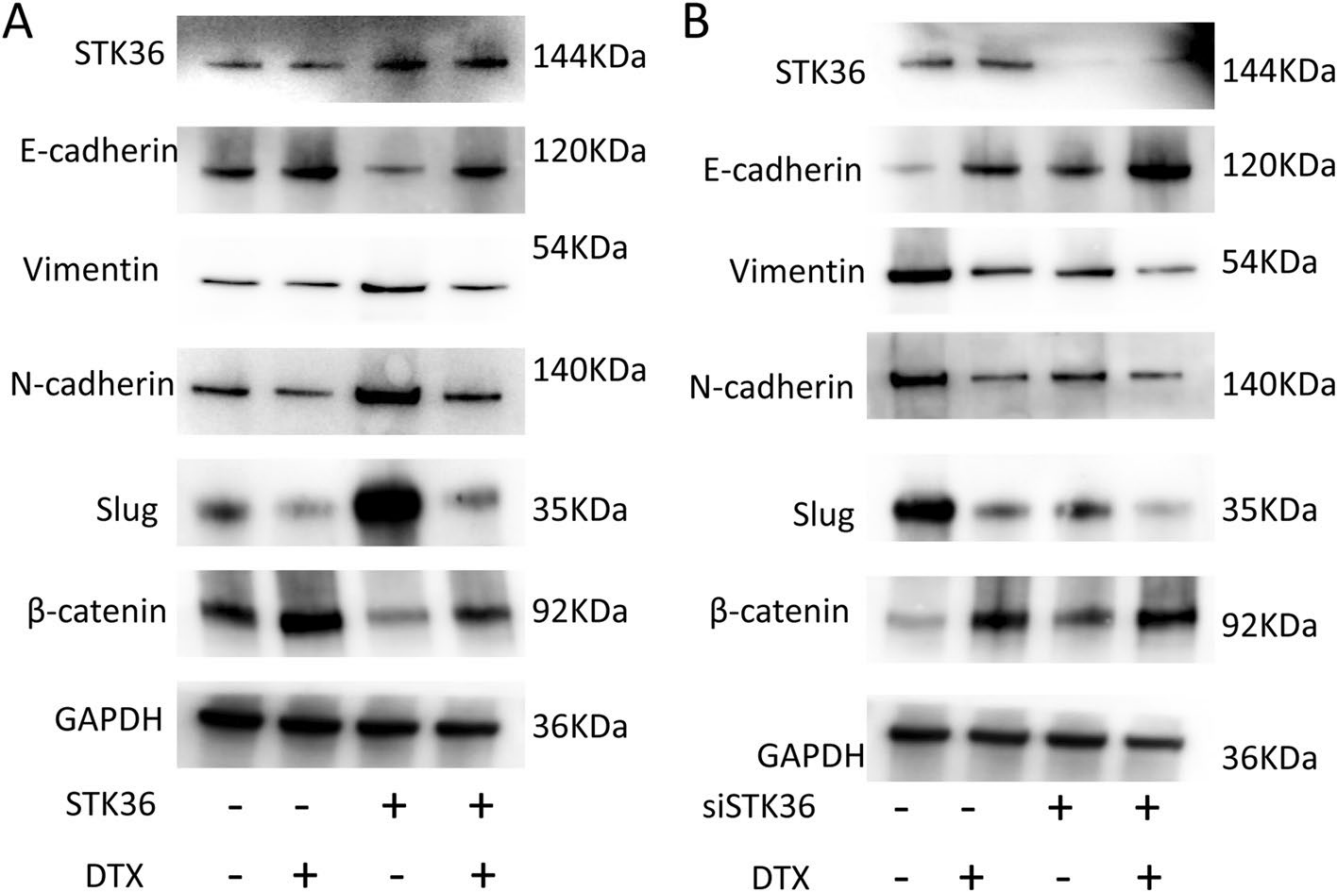
STK36 reduces the sensitivity of PCa cells to docetaxel by regulating epithelial-mesenchymal transition. **(A, B)** The docetaxel-treated concentration for PC-3 was 0.1 μmol/L, while it was 5 nM for DU-145. STK36 was overexpressed (**A**) or sliced (**B**) in DU-145 cells, and EMT markers were detected.

To explore the potential relationship between STK36 and docetaxel resistance in PCa, expression profiles of some factors in EMT were detected. When STK36 was overexpressed, the expression of E-Cadherin, and β-Catenin was decreased, while Vimentin, N-Cadherin, and Slug levels were improved (Fig. 7). An opposite action was seen after STK36 knock-down; the expression of E-Cadherin and β-Catenin increased while Vimentin, N-Cadherin, and Slug decreased (Fig. 7).

## Discussion

Docetaxel is a potent cytotoxic drug in the treatment of advanced PCa. Still, some cases tend to have poor initial response to docetaxel-based therapy. In this study, we assessed the role and potential mechanism of STK36 in docetaxel resistance-PCa.

Previous studies found that STK36 can intervene in the response of glioblastoma multiforme to temozolomide and a pathological variant of gastric cancer^29,30^. In this study, we used tissue microarray and different databases to explore the role of STK36 and PCa. Considerably higher STK36 levels were found in patient tissues compared to benign. Given that the STK36 could accept the hedgehog signal as a vertical signaling component, the expression profile variation gives a huge possibility of ligation to the peculiarity of PCa^31^. To simulate docetaxel resistance mechanisms that might occur in humans, a model based on two sensitive cells, DU-145 and PC3, was established and used in this study^32^. Overexpression of STK36 in these cells improved cell proliferation, invasion, and migration, reducing the docetaxel sensitivity. However, the cell apoptosis was not significantly impacted, which might be due to the low apoptosis property of tumor cells^33^. Once cell apoptosis was accelerated with docetaxel treatment, the avail of STK36 became visible. The role of STK36 was further confirmed by STK36 overexpression, which reversed the above effects.

The reactivation of EMT has an important role in cancer progression. It can confer metastatic properties to tumor cells^34,35^. The first molecular range for activation of EMT is the reduced E-cadherin levels, which is responsible for lateral contact of epithelial cells through adherens junctions^36^. In this study, reduced E-cadherin and increased N Cadherin and Vimentin levels were observed in cells overexpressing STK36, which suggested an interaction between STK36 and EMT. EMT can accelerate the motility and migration of cancer cells besides their morphological alteration^37,38^. The CCK8 and cell wound scratch assay confirmed the claim.

Many factors are involved in triggering EMT, including upstream mediators such as Snail, Slug, ZEB proteins, and transforming growth factor-beta (TGF-β)^39,40^. Moreover, mesenchymal markers are not confined to N-cadherin, vimentin, fibronectin, α-smooth muscle actin (SMA), and matrix metalloproteinases (MMPs)^41,42^. Therefore, EMT is a major existing signaling that bridged the STK36 and PCa.

In PCa, circRNA FOXO3 can promote the expression of FOXO3 to inhibit EMT, leading to docetaxel sensitivity^43^. ZEB1 is a critical inducer of EMT in cancer, and the interaction between ZEB1 and EMT can lead to drug resistance^44^. Therefore, EMT induction is vital for promoting docetaxel resistance. The former report indicated that the importance of EMT was enhancing the survival and viability of cancer cells upon exposure to docetaxel^45^, which is probably the basis that STK36 impacted the docetaxel resistance in PCa. However, the real process and mechanism of EMT mediated the STK36 interfering with the docetaxel resistance in PCa must be more complex. Exosomal transfer of miRNA-200c in tongue squamous cells could lead to EMT inhibition and enhance the sensitivity of cancer cells to docetaxel chemotherapy^46^. Upon docetaxel administration, EMT induction occurred in PCa cells; docetaxel sensitivity could be influenced using pomolic acid^47^.

In summary, our data suggested that overexpression of STK36 has a force-tumor effect in models of docetaxel-sensitive PCa. Up-regulation of STK36 markedly enhances cell proliferation, invasion, and migration and reduces docetaxel sensitivity to PC-3 and DU-145 cells. The docetaxel sensitivity variation was probably resulting from the activation of EMT. This study found that STK36 has an impact on various biological phenotypes of prostate cancer cells. Due to time and funding constraints, this article mainly conducts in-depth research on the mechanism of STK36 and docetaxel chemotherapy resistance. The detailed mechanisms related to cell promotion capacity, migration capacity, and invasive capacity have not been fully analyzed This is the inadequacy of this article. Whether STK36 is a good candidate target in clinical trials and can be used as an adjuvant for the treatment of docetaxel resistant prostate cancer with docetaxel requires further in vivo confirmation, which is also our next research direction.

### Supplementary Information

Supplementary Information 1.

Supplementary Information 2.

## Data Availability

The datasets used and/or analyzed during the current study are available from the corresponding author upon reasonable request.
