# Supplementary figures and images for: Serine/threonine kinase 36 induced epithelial-mesenchymal transition promotes docetaxel resistance in prostate cancer
Source: Sci Rep. 2024 Jan 6;14:729. doi: 10.1038/s41598-024-51360-9 (PMC10771505; doi:10.1038/s41598-024-51360-9)

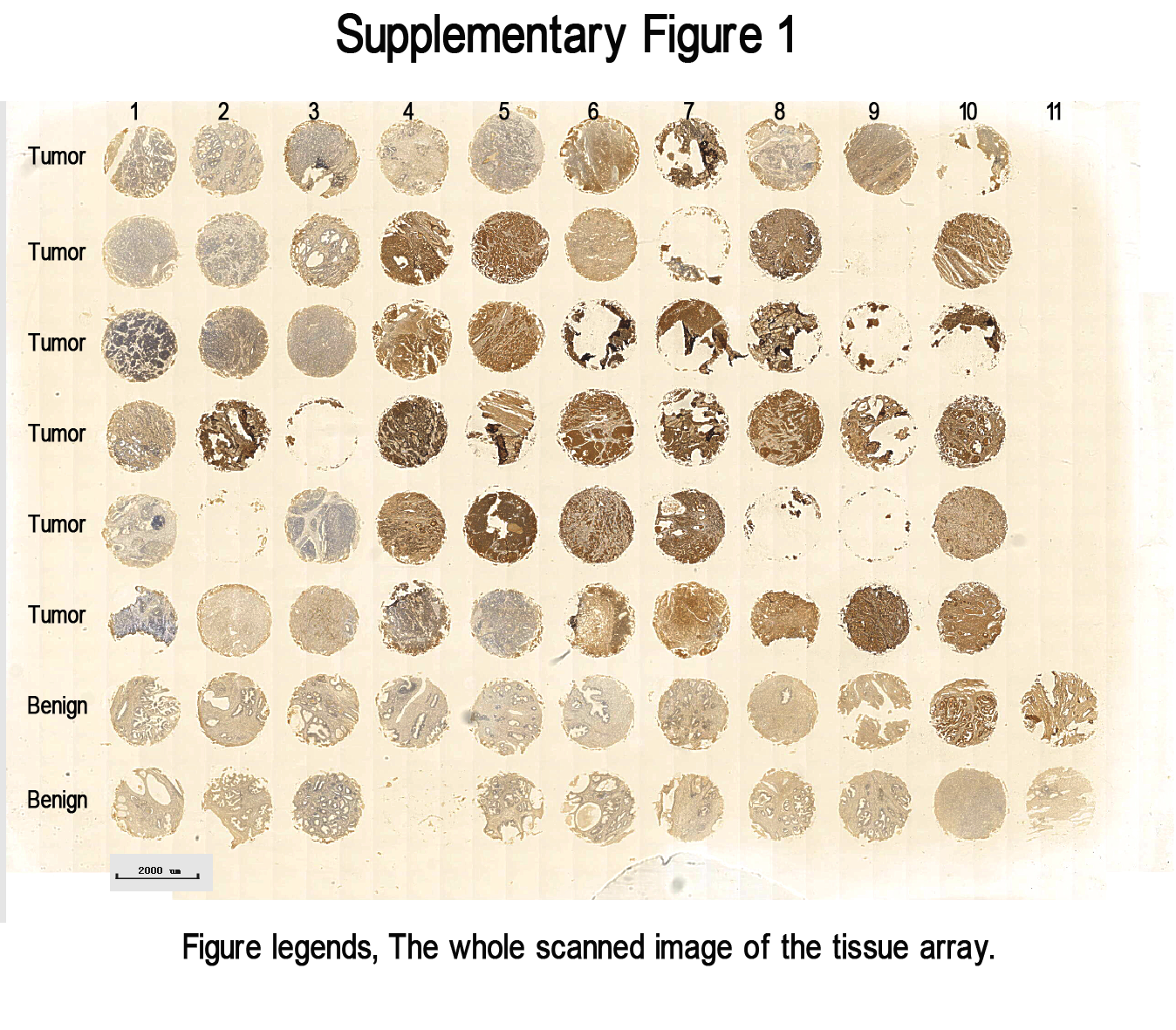

Supplement: Supplementary file 1 — Supplementary Information 1. [file 41598_2024_51360_MOESM1_ESM.tif]
