# Supplementary material for: Serine/threonine kinase 36 induced epithelial-mesenchymal transition promotes docetaxel resistance in prostate cancer
Source: Sci Rep. 2024 Jan 6;14:729. doi: 10.1038/s41598-024-51360-9 (PMC10771505; doi:10.1038/s41598-024-51360-9)

The docetaxel-treated concentration for PC-3 was 0.1 μmol/L, while it was 5 nM for DU-145. STK36 overexpression was detected by Western blotting. The bands shown in revised Figure 2A

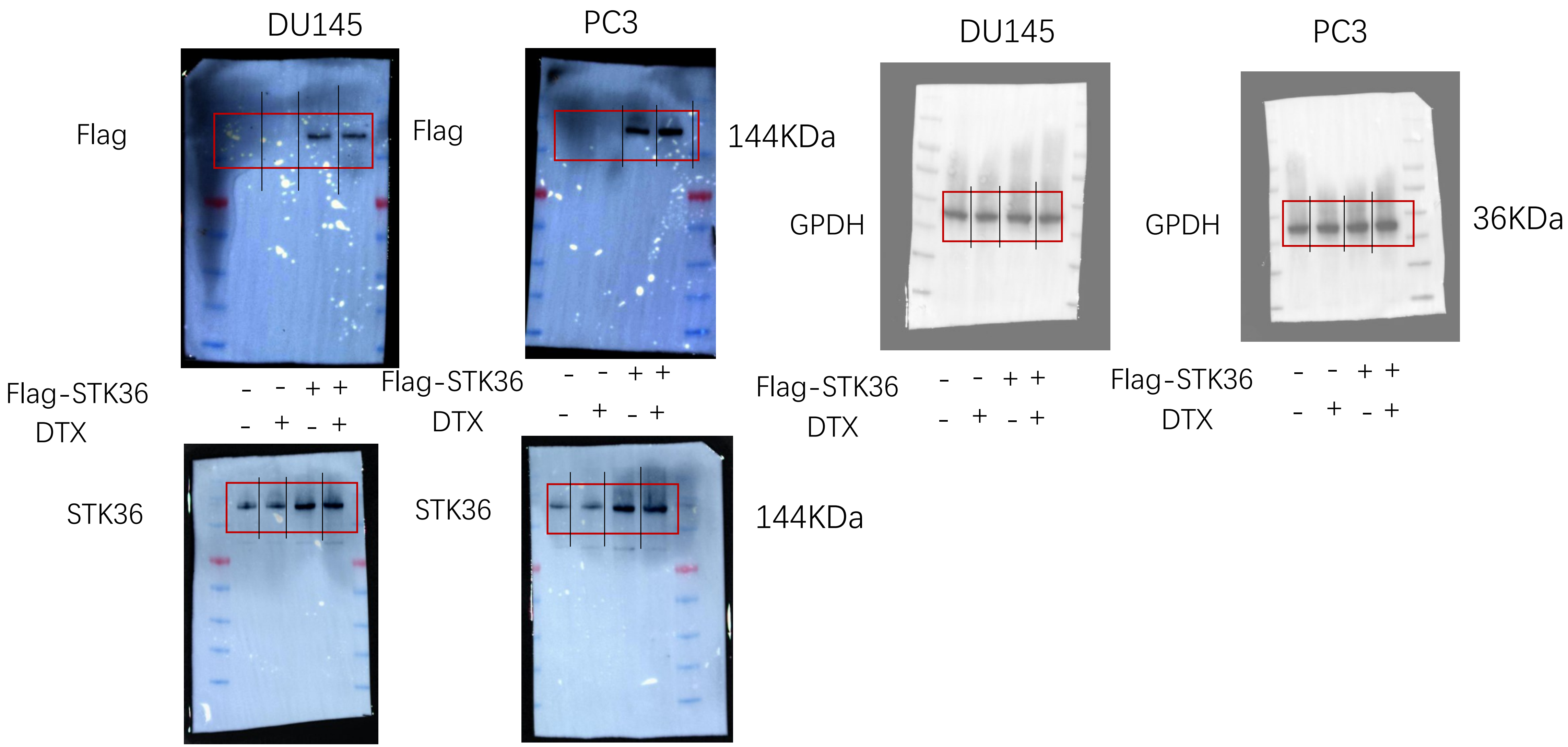

Figure4 The docetaxel-treated concentration for PC-3 was 0.1 μmol/L, while it was 5 nM for DU-145. STK36 overexpression was detected by Western blotting.

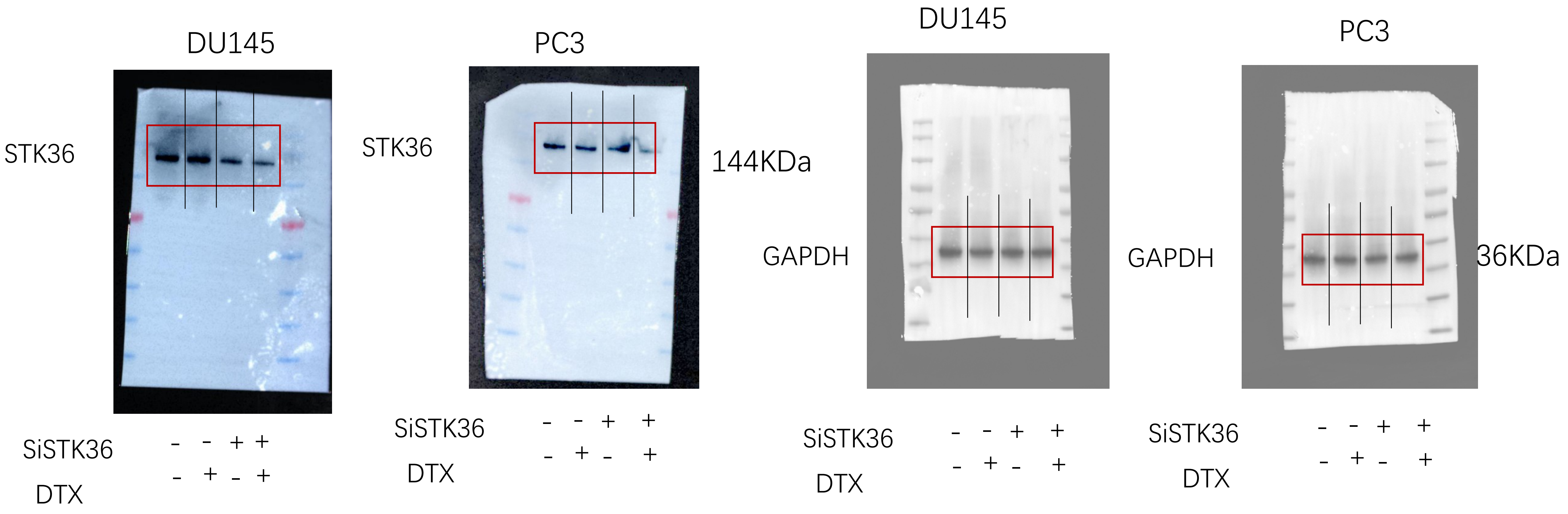

Figure 7 The docetaxel-treated concentration for PC-3 was 0.1 μmol/L, while it was 5 nM for DU-145. STK36 was overexpressed (A) in DU-145 cells, and EMT markers were detected.

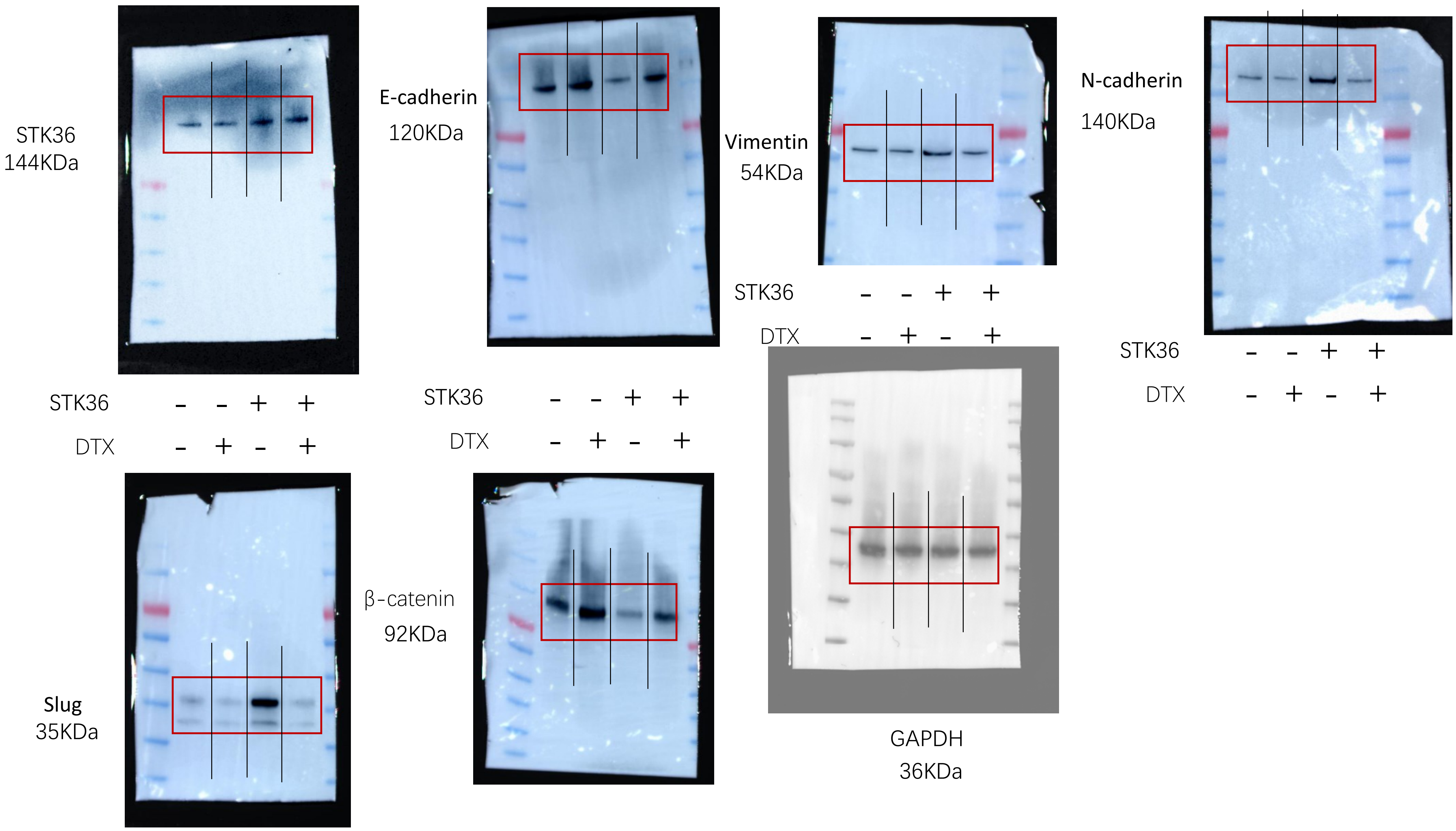

Figure 7 The docetaxel-treated concentration for PC-3 was 0.1 μmol/L, while it was 5 nM for DU-145. STK36 was sliced (B) in DU-145 cells, and EMT markers were detected.

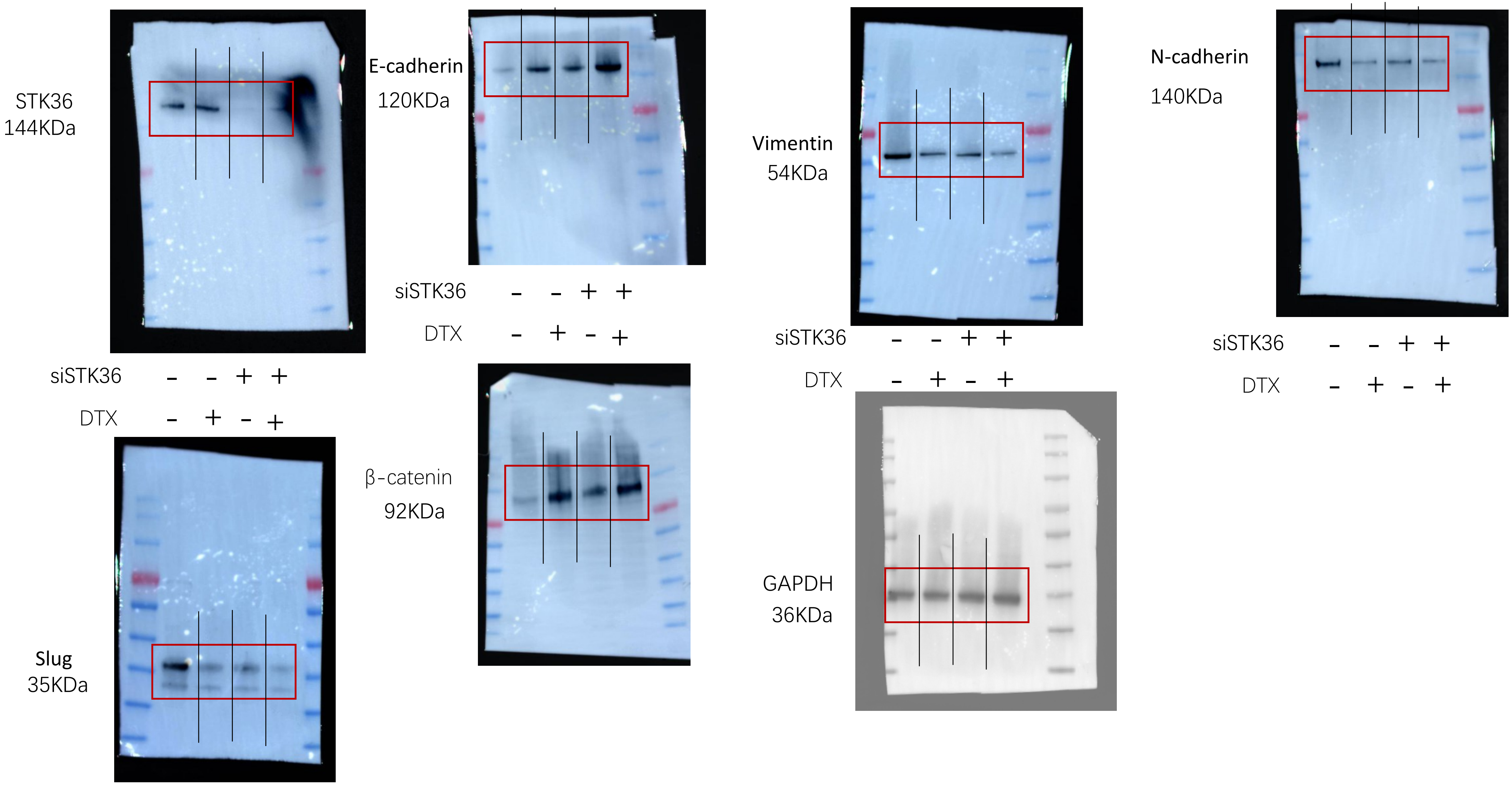

Supplement: Supplementary file 2 — Supplementary Information 2. [file 41598_2024_51360_MOESM2_ESM.pdf]
